# A WDR Gene Is a Conserved Member of a Chitin Synthase Gene Cluster and Influences the Cell Wall in *Aspergillus nidulans*

**DOI:** 10.3390/ijms17071031

**Published:** 2016-06-29

**Authors:** Gea Guerriero, Lucia Silvestrini, Michael Obersriebnig, Jean-Francois Hausman, Joseph Strauss, Inés Ezcurra

**Affiliations:** 1 Department of Applied Genetics and Cell Biology, Fungal Genetics and Genomics Unit, University of Natural Resources and Life Sciences Vienna (BOKU), Bioresources and Technologies Campus Tulln-Technopol, Tulln/Donau A-3430, Austria; lucia.silvestrini@boku.ac.at (L.S.); joseph.strauss@boku.ac.at (J.S.); 2 Environmental Research and Innovation (ERIN), Luxembourg Institute of Science and Technology (LIST), Esch/Alzette L-4362, Luxembourg; jean-francois.hausman@list.lu; 3 Institute of Wood Science and Technology, University of Natural Resources and Life Sciences Vienna (BOKU), Bioresources and Technologies Campus Tulln-Technopol, Tulln/Donau A-3430, Austria; m.obersriebnig@boku.ac.at; 4 Health and Environment Department, Austrian Institute of Technology GmbH—AIT, University and Research Center Campus Tulln-Technopol, Tulln/Donau A-3430, Austria; 5 KTH, School of Biotechnology, Albanova, Stockholm SE-10691, Sweden

**Keywords:** *Aspergillus nidulans*, WDR gene, beta-flanking gene, chitin synthase, collinear genes, cell wall

## Abstract

WD40 repeat (WDR) proteins are pleiotropic molecular hubs. We identify a WDR gene that is a conserved genomic neighbor of a chitin synthase gene in Ascomycetes. The WDR gene is unique to fungi and plants, and was called *Fungal Plant WD* (*FPWD*). *FPWD* is within a cell wall metabolism gene cluster in the Ascomycetes (Pezizomycotina) comprising *chsD*, a Chs activator and a GH17 glucanase. The *FPWD*, AN1556.2 locus was deleted in *Aspergillus nidulans* strain SAA.111 by gene replacement and only heterokaryon transformants were obtained. The re-annotation of *Aspergilli* genomes shows that AN1556.2 consists of two tightly linked separate genes, i.e., the WDR gene and a putative beta-flanking gene of unknown function. The WDR and the beta-flanking genes are conserved genomic neighbors localized within a recently identified metabolic cell wall gene cluster in genomes of *Aspergilli*. The heterokaryons displayed increased susceptibility to drugs affecting the cell wall, and their phenotypes, observed by optical, confocal, scanning electron and atomic force microscopy, suggest cell wall alterations. Quantitative real-time PCR shows altered expression of some cell wall-related genes. The possible implications on cell wall biosynthesis are discussed.

## 1. Introduction

In eukaryotes, WD40-repeat (WDR) proteins are often found in protein complexes and may participate in different cellular pathways, a phenomenon sometimes referred to as moonlighting. WDR proteins contain conserved GH and WD residues (hence their name) and their most widespread structure is a seven-bladed β-propeller [1]. Several WDR proteins have been characterized in fungi, where they are involved in different processes, namely cell differentiation [2], vegetative incompatibility [3], nuclear migration [4], mating and virulence [5]. WDR proteins are also involved in processes related to the cell wall. For example, a WDR protein, RACK1, primarily involved in mating and virulence in *Ustilago maydis*, regulates cell wall integrity [5] and deletion of the nuclear migration gene *nudC* in *Aspergillus nidulans*, which codes for a WDR protein, causes an abnormal deposition of chitin in vacuoles [4].

Previous results showed conserved genomic microsynteny and partial co-expression of a glycosyltransferase from family 2 (GT2, i.e., a cellulose synthase, *CesA*) and a WDR gene in plants [6,7], and that the collinear arrangement of genes coding for a GT2 enzyme and a WDR protein is frequent in eukaryotes’ genomes. Here, we show that conserved collinearity of a GT2-WDR gene pair is also present in fungi, where a gene coding for a WDR protein is a conserved gene neighbor of a class IV chitin synthase (*chsD*). Recently, a comprehensive in silico analysis of genomic blocks centered on class IV *chs* identified the presence of a cell wall metabolism gene cluster in *Aspergillus* [8]. We show that the WDR gene is located within this *Aspergillus* cell wall metabolism gene cluster. The role of physically clustering functionally related genes in eukaryotes is unknown, but could involve coordinated gene expression or keeping genes together to avoid toxic effects of their individual deletion. To shed light on the role of this *A. nidulans* WDR gene, a knock-out mutant was produced by gene replacement of the AN1556.2 gene locus, which contains the WDR gene and a tightly linked small beta-flanking (*bf*) gene that was identified upon later annotation. Deletion of AN1556.2 causes cell-wall related phenotypes, but it remains to be shown whether these are caused by the WDR gene, the *bf* gene, or both.

## 2. Results

### 2.1. A WDR Gene Is a Conserved Neighbor of chs Genes in Fungi

**Figure 1:**
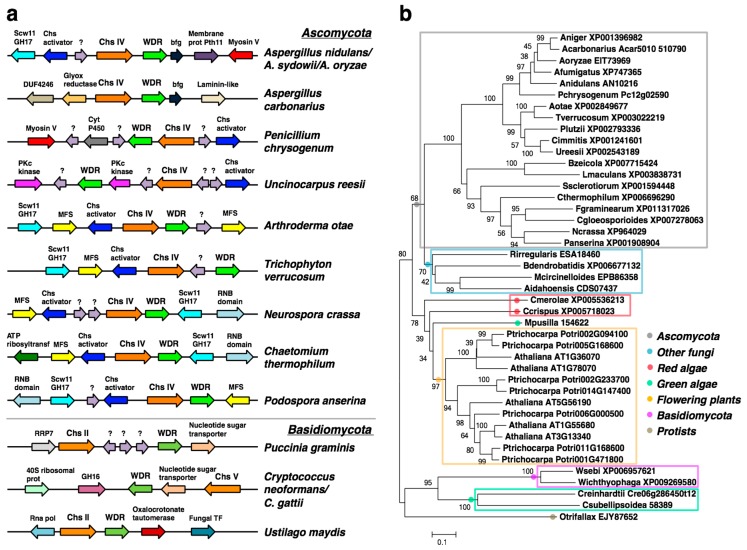
Genomic collinearity *chs*-WDR and phylogeny of FPWD. (**a**) Cartoon showing conserved genomic association between a *chs* and a WDR gene in a representative set of fungi. Lilac arrows with question marks indicate hypothetical proteins with no described function. Legend: GH, glycosylhydrolase; Chs, chitin synthase; RNB, ribonuclease II; Cyt P450, cytochrome P450; DUF3435, domain of unknown function 3435; MFS, major facilitator superfamily transporter; bfg, beta-flanking gene; Glyox reductase, glyoxylate reductase; (**b**) FPWD homologs in fungi and extant taxa. The protein sequences used to build the tree are indicated in the figure. Number of bootstraps = 1000. Scale bar, evolutionary distance of 0.1 amino acid substitutions per position.

Bioinformatic analyses identified a fungal WDR gene that is a conserved collinear gene neighbor of class IV *chs*, *chsD*, in Ascomycota (subdivision Pezizomycotina), and is represented by AN10216 in *Aspergillus nidulans* (Figure 1a and Table S1). A collinear *chs*-WDR gene arrangement is also detected in some basidiomycetes (Figure 1a), although in these taxa both the *chs* and the WDR genes belong to distinct subfamilies. Thus, whereas the *chs* genes are of either class II (*Ustilago maydis* and *Puccinia graminis*) or class V (*Cryptococcus*), the WDR gene of *Cryptococcus gattii* and *C. neoformans* belong to the WDR89 family, and the WDR genes of *P. graminis* and *U. maydis* belong to the WDR36 and WDR75 family, respectively. Figure 1a shows that the WDR gene is within a cluster of conserved gene neighbors in the Ascomycetes (Pezizomycotina) comprising *chsD*, a Chs activator and a GH17 glucanase. These latter genes were recently identified as members of a cell wall metabolism gene cluster in *Aspergillus* [8].

BLAST and phylogenetic analyses revealed that the closest homologues of AN10216 outside of fungi are in plants, and that there are no AN10216 homologs in metazoans. Therefore the AN10216 gene product was named Fungal-Plant WDR, FPWD. The *FPWD* gene is found as a single gene in fungal species, and mostly in the Ascomycota (Figure 1b). It is also found in other fungal taxa, but appears to be mostly absent in Basidiomycota, with exception of few species. In the Kingdom Plantae, it is found in land plants, green algae and red algae. In flowering plants, it occurs as a small gene family of five closely related genes, in *Arabidopsis thaliana* represented by AT1G36070, AT1G78070, AT1G55680, AT3G13340 and AT5G56190. The plant genes group into two monophyletic clusters, suggesting their functional conservation (Figure 1b). Finally, outside of Fungi and Plantae, FPWD is found in diverse marine protists, indicating its ancient evolutionary origins.

Examination of their expression using publicly available microarray databases in *Arabidopsis* and poplar (BAR eFP) suggests that members in one cluster are mainly xylem-specific, whereas members in the other cluster are largely pollen-specific (Table S2). This is consistent with a possible role for the plant FPWDs in the cell wall processes, because both formation of the secondary cell wall or the pollen tube are processes involving extensive cell wall synthesis.

**Figure 2:**
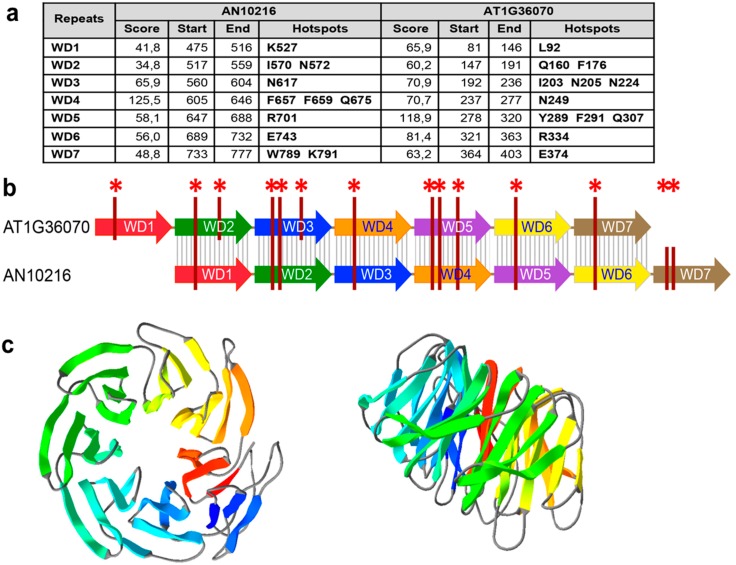
Predicted 3D structure, WD blade details and interaction hotspots of FPWD. (**a**) Prediction of WD repeats in AN10216 (aa 423–827) and AT1G36070, showing residues predicted to be involved in the top face protein–protein interaction. Similar results were obtained when other plant FPWDs were analyzed. The accuracy score of the prediction is 89% ± 5%; (**b**) WD repeats are shifted between AN10216 and AT1G36070. Grey stripes indicate sequence similarity. Vertical lines with asterisks indicate the hotspots in (**a**); (**c**) Front (**right**) and lateral (**left**) view of the predicted 3D structure of AN10216 (aa 423–764).

Identification of WD repeats predicted seven repeats in both fungal and plant FPWDs (Figure 2a), and revealed that the hotspots of protein–protein interaction on the top face are conserved between fungi and plants, although the WD repeats are shifted between the two Kingdoms (Figure 2b). Structure prediction confirmed a seven-bladed propeller (Figure 2c).

### 2.2. Deletion of the AN1556.2 Locus Alters Growth and Cell Wall Properties

**Figure 3:**
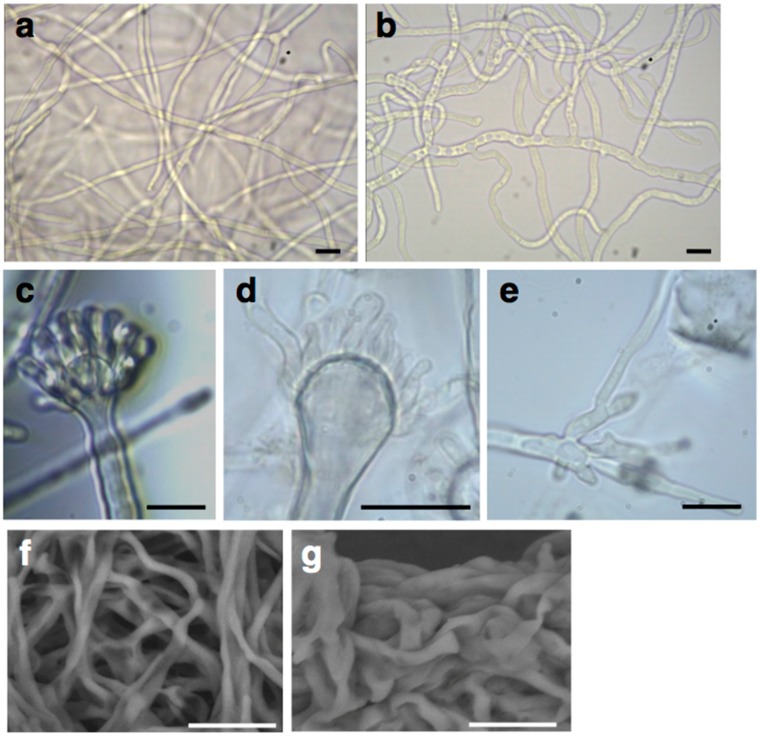
Phenotypes of the *FPWD* replacement mutant, hkΔAN1556, and wild-type control strain SAA.111: (**a**) SAA.111 hyphae; (**b**) hkΔAN1556 curly hyphae; (**c**) SAA.111 conidiophore; (**d**,**e**) aberrant conidiophores of hkΔAN1556; and (**f**,**g**) SEM images of control and hkΔAN1556, respectively, grown in liquid medium. Bars refer to 10 µm in (**a**–**e**) and to 20 µm in (**f**,**g**).

The whole AN1556.2 locus containing the *FPWD* gene, as well as a tightly linked small beta-flanking (*bf*) gene that was identified upon later annotation, was deleted by gene replacement (hence both *FPWD* and *bf* genes were deleted). Transformation of protoplasts with the replacement cassette resulted in only 6 independent transformants. When grown on fresh solid media, five of these were phenotypically identical to SAA.111 (the recipient strain), while one was displaying strongly delayed growth. This transformant was further characterized by Southern blotting and PCR (Figure S1), revealing the presence of a heterokaryotic mycelium carrying nuclei with intact copies of the gene and nuclei carrying the deletion of the locus. The heterokaryotic strain (designated hkΔAN1556) showed vacuolated and wavy hyphae (Figure 3b). Moreover, it differentiated aberrant conidiophores, which failed to develop normal metulae (Figure 3d) and vesicles (Figure 3e). When grown in liquid medium (minimal medium without agar), hkΔAN1556 formed large irregularly-shaped clumps, a feature which was not observed in the control (i.e., the SAA.111 strain) (Figure S2). SEM observations confirmed the presence of clumped hyphae in hkΔAN1556 (Figure 3g), suggesting cell wall anomalies.

**Figure 4:**
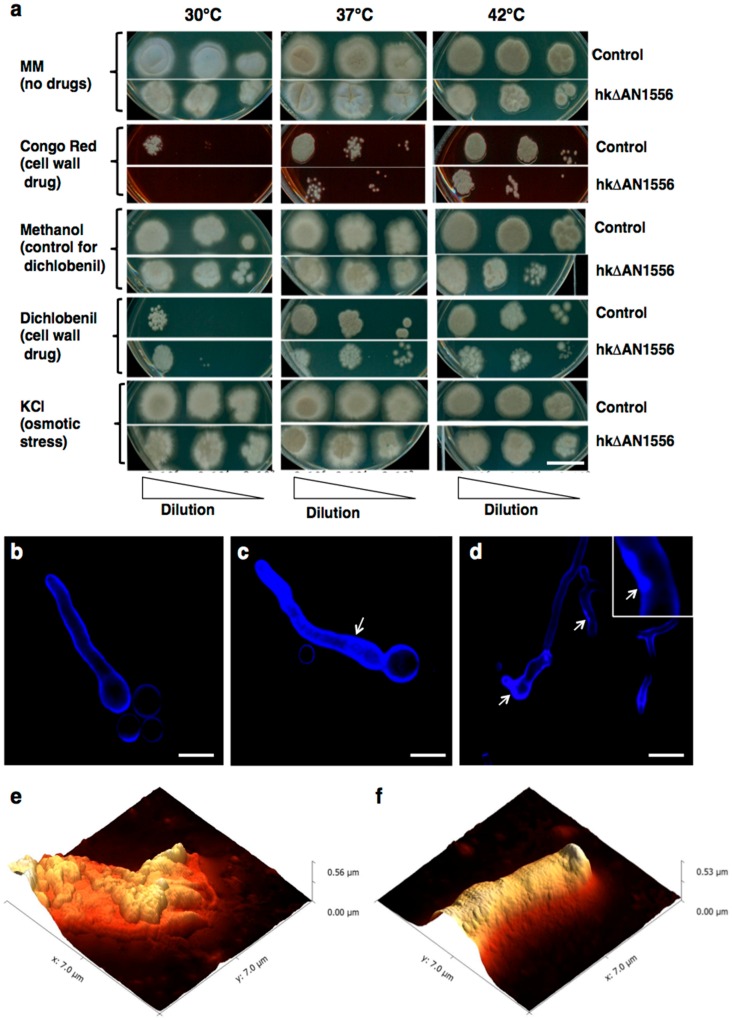
Cell wall-related phenotypes of *FPWD* replacement. (**a**) Cell wall drug sensitivity of hkΔAN1556 and control SAA.111. Growth test in the presence of cell wall drugs (CR 100 µM and DCB 200 µM) and osmotic stress. Different concentrations of spore suspension were analyzed (10^6^, 10^4^, 10^2^). Bar equals 1 cm; (**b**–**d**) Confocal microscope pictures of control (**b**) and hkΔAN1556 (**c**,**d**) stained with Calcofluor White (CFW). Arrows indicate fluorescent granules accumulating along the hyphae. Inset picture in panel **d** shows detail of CFW granules. Bars equal 5 µm; (**e**,**f**) Ultrastructural features of hkΔAN1556; Representative AFM images of control (**e**) and hkΔAN1556 (**f**) showing 3D height with enhanced topographic effect arising from shadows after virtual illumination.

To further investigate the impact of AN1556.2 deletion on the cell wall, growth tests were carried out with the cell wall drugs Congo Red (CR) and dichlobenil (DCB) [9]. As Figure 4 shows, hkΔAN1556 is highly sensitive to CR, a drug that targets chitin, at all temperatures. DCB also inhibits growth of hkΔAN1556 at higher temperatures, although at a lower temperature (30 °C) the opposite effect is observed. Osmotic stress (KCl 0.5 M) only slightly affects growth of hkΔAN1556.

Confocal microscopy using Calcofluor White (CFW) as a chitin dye shows higher fluorescence in hkΔAN1556 (Figure 4c), and accumulation of particles, which could be chitin granules, was detected along the hyphae (Figure 4d, arrows), suggesting altered chitin synthesis. Studies by AFM revealed differences in ultrastructural topography: while the control strain appears rougher, with subunits that are more heterogeneous in size and distribution, the surface of hkΔAN1556 hyphae is smoother, with regular subunits (Figure 4e,f).

### 2.3. Expression of Cell Wall-Related Genes

**Figure 5:**
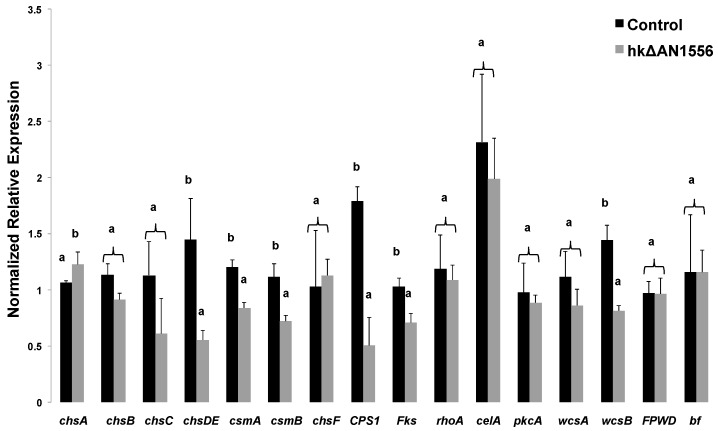
Gene expression analysis of cell wall-related genes in hkΔAN1556 and in the control SAA.111. Different letters indicate statistically different values (*p* < 0.05).

Cell wall disturbance alters gene expression in both plants [10] and fungi [11]. We therefore examined the expression of several cell wall-related genes: the target genes were the *chs* genes, the putative β-1,3;1,4 glucan synthase *celA*, the β-1,3-glucan synthase *fksA* and the Rho-related GTPase *rhoA* [12], together with three wall sensors (*pkcA*, *wscA* and *wscB*; [13,14,15,16]) and a putative capsule polysaccharide synthase (CPS1) which shows strong sequence homology to hyaluronan synthase. Whereas *chsA* showed increased expression, the majority showed decreased expression in hkΔAN1556 (Figure 5). In particular the wall sensor *wscB* shows a statistically significant decrease in the transformed strain. Although the other two genes involved in sensing cell wall integrity in *A. nidulans* (i.e., *pkcA* and *wscA*) do not show statistically significant differences, their pattern can be interpreted as a trend towards decrease (Figure 5). Intriguingly, the expression levels of *FPWD* and *bf* in hkΔAN1556 are comparable to the control, in spite of the presence of nuclei in the heterokaryons carrying a deletion of this locus (Figure 5). This suggests compensatory expression in nuclei where these genes are intact.

### 2.4. Genetic Analyses of hkΔAN1556

**Figure 6:**
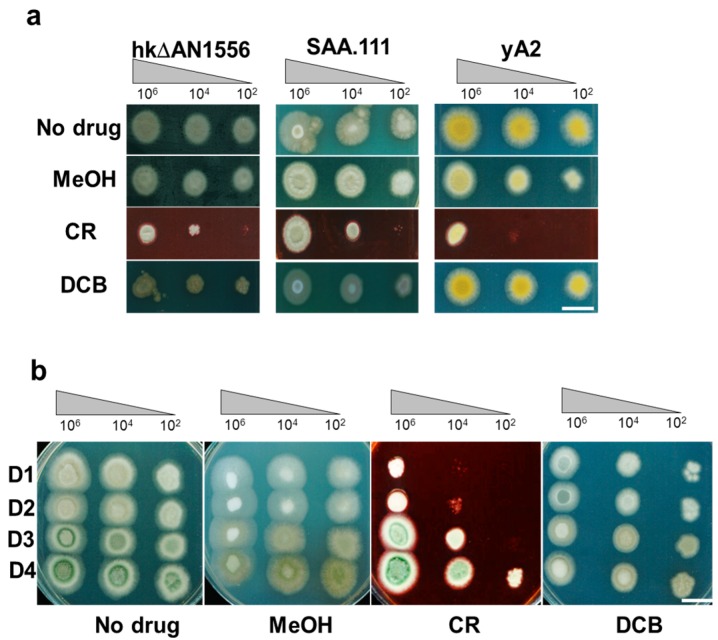
Sensitivity of diploids (four independent strains, indicated D1, D2, D3 and D4) to cell wall drugs: (**a**) Different concentrations of spore suspension of the hk∆AN1556, the controls SAA.111, and yA2; and (**b**) the diploids were analyzed (10^6^, 10^4^, and 10^2^). The strains were incubated at 37 °C for two days. MeOH is methanol (control for DCB), CR is Congo Red and DCB is dichlobenil. Bars refer to 1 cm.

Because only heterokaryon transformants were recovered, the viability of a homokaryotic strain was checked. After several rounds of sporulation and low-density plating of the resulting conidia, PCR on genomic DNA and Southern blotting on the obtained six colonies showed that homokaryons could not be obtained. This suggests that the deletion of the *FPWD* and *bf* genes is lethal. To test whether the hkΔAN1556 phenotype is truly dominant or rather represents a dose effect of an unbalanced heterokaryon, stable diploids of hkΔAN1556 were produced by crossing the heterokaryon with strain yA2 [17], using established genetic methods (see Experimental section). Several independent diploid colonies were recovered (hereafter referred to as D1–D4), and these were phenotypically identical to hkΔAN1556, and showed the same growth characteristics (non-straight vacuolated hyphae) in liquid media (Figure S3). Growth tests on solid media supplemented with cell wall drugs showed that hk∆AN1556 is more resistant to CR, while slightly more susceptible to DCB (Figure 6a) than yA2 and these traits are present in the diploids (Figure 6b). Indeed, the diploids show a response to cell wall drugs that is more similar to hk∆AN1556. It should be noted that, among the four diploids, D4 shows higher resistance to CR at higher spore dilutions than the other three diploids (Figure 6b). Further studies are necessary to determine whether this difference is due to epigenetic events. Taking together, the hyphae phenotype of the diploids and the results of the growth tests on solid media, it can concluded that the genes contained in the AN1556.2 locus are essential and that its deletion of this locus results in a dominant cell wall-related phenotype.

### 2.5. Bioinformatic Analyses of the bf Gene in the AN1556.2 Locus

The re-annotation of *Aspergilli* genome carried out by Aspergillus Genome Database curators using PASA (Program to Assemble Spliced Alignments) analysis [18,19], indicated that the AN1556.2 locus consists of two separate neighboring genes, *FPWD* (AN10216) and a closely linked putative beta-flanking gene, AN10219 (*bf*), which is the homolog of a gene of unknown function flanking the A mating-type locus in Basidiomycetes [20].

We verified that *FPWD* (AN10216) and *bf* (AN10219) truly are two separate genes by PCR of cDNA (Figure S4). The association between *FPWD* and the *bf* gene is conserved in the genus *Aspergillus* (Table S3), although the function of the *bf* gene is unknown.

The Bf proteins lack homologs outside fungi, contain Gly-rich stretches (Figure S5), and are highly disordered as predicted by computational analysis using CSpritz [21]. Intrinsically disordered proteins may stabilize protein complexes by binding to partners through short linear motifs (SLiMs) and computational analysis using ELM [22] identified several potential SLiMs, including a fungal variant of the WDR5-binding motif that mediates assembly of protein complexes involved in histone modification. Both the intrinsic disorder and the WDR5-binding SLiM are predicted in all *Aspergillus* Bf proteins.

## 3. Discussion

We show that *FPWD*, a gene coding for a WDR protein, is a conserved collinear genomic neighbor of *chsD* in Ascomycetes. The *chsD* and *FPWD* genes, together with a closely linked *bf* gene, are located within a cell wall metabolic gene cluster recently identified in *Aspergilli* [7]. The study identified the fungal wall gene cluster based on the predicted functions of conserved gene neighbors of *chsD*, which comprise a Chs activator, a myosin V, a GH17 cell wall glucanase, scw11, a serine/threonine kinase, and a type 2A protein phosphatase PP2A. The authors propose that the myosin V possibly enables vesicle transport of chitin synthase D, whereas the kinase and PP2A possibly regulate myosin V activity [7]. In Figure 1a, we show the cluster’s core members in *A. nidulans*, *A. oryzae* and *A. sidowii*, namely *chsD*, the Chs activator, the myosin, and the GH17 glucanase (the PP2A and the kinase are not shown and lie barely outside of the depicted region). Our analysis establishes that the fungal wall gene cluster is present in several classes within the Pezizomycotina in Ascomycetes, and that the conserved core members comprise *chsD*, *FPWD*, the Chs activator and the GH17 glucanase, thus identifying *FPWD* as a plausible cell wall-active gene. Also, the analysis identifies a potential novel functional member, namely the MFS transporter, which is a highly conserved gene neighbor within the cluster in several species, and could function in transporting sugars across membranes. FPWD belongs to the category of seven-bladed propeller proteins, and is structurally similar to the coatomer subunit alpha, as computed by Phyre2 (Fold library id c5a1vK_).

Our study also provides experimental evidence suggesting a role of FPWD in the cell wall. Deletion of *FPWD* and the closely linked *bf* gene resulted in heterokaryon transformants (hkΔAN1556) displaying cell wall-related phenotypes. Notably the transformants had morphological defects and formed clumps when grown in liquid media (Figure 3g), showed increased sensitivity to cell wall-perturbing agents (Figure 4a), had smaller surface subunits (Figure 4f), and displayed altered expression of wall-related genes (Figure 5). Put together, the close genomic vicinity of fungal *FPWD* to *chsD* in Ascomycetes, its localization within a cell wall metabolic gene cluster in *Aspergilli*, and the mutant phenotypes, suggest its role in the cell wall, although its exact function is unknown. Because WDRs are known to act as protein scaffolds, fungal FPWDs could participate as a hub in the assembly of chitin biosynthesis complexes, or in their vesicle trafficking. The closely linked *bf* gene was within the deleted locus in the mutant and thus the deletion phenotypes could be caused by removal of *FPWD*, *bf*, or both. The *bf* genes localize to the cell wall metabolic gene cluster in *Aspergilli* (Table S3), also suggesting a role in the cell wall. Bfs belong to the category of intrinsically disordered proteins and have short amino acid motifs that resemble WDR5-binding SLiMs, so it could possibly bind to FPWD.

The phenotype of the heterokaryotic transformants is dominant and its dominance was confirmed in diploids (Figure 6). A plausible explanation invokes the presence of nuclei occupying cytoplasmic regions with distinct properties within the *A. nidulans* hyphae, a phenomenon that was observed in syncytial hyphae of *Ashbya gossypii* heterokaryons carrying deletions in genes involved in mitochondrial fusion/fission [23].

Our results suggest that identifying conserved gene neighbors may be a useful bioinformatic approach to mining for gene functions, in addition to more traditional approaches based on gene expression and proteomics. Although metabolic gene clusters are emerging as common in both fungi and plants [24], only a recent bioinformatic analysis revealed such an arrangement in fungal cell wall biosynthesis [7], and we provide experimental evidence for a role in the cell wall of *FPWD*, a gene within the cluster that lacked a predicted wall-related function. Recently, a plant WDR protein, TWD40-2, was linked to clathrin-mediated endocytosis of cellulose synthases during cellulose biosynthesis, and is thus the first plant wall-related WDR [25]. In plants, cell wall genes are generally scattered in the genome, but we noticed that the *TWD40-2* genes are single-copy in angiosperm genomes, and their genomic neighbors comprise genes with known or potential roles in the cell wall, such as TBL21 (acetylation of wall polymers), CTP synthase (UDP-glucose metabolism), Peptidase M50 (ER stress), ABC transporter, cathepsin B (delays PCD), Man5-7 (hydrolysis of cell wall polymers) and cycloartenol synthase (sterol biosynthesis). Further, the genomic neighbors of plant *FPWDs* also comprise genes with known or potential roles in the cell wall, such as trehalose-6-phosphate phosphatase (trehalose metabolism), GH3 hydrolase and actin ACT7 (in higher plants), as well as nucleotide sugar transporter and GT57 glucosyltransferase (in green algae). The precise functions of fungal and plant cell wall-related WDRs, and of their potentially wall-involved gene neighbors, remain to be further elucidated.

## 4. Experimental Section

### 4.1. Bioinformatic Analyses of AN10216

The search for AN10216 homologs in other *Aspergilli* was carried out using the BLAST+ suit (BLASTP algorithm) at the AspGD multi-genome search website (http://www.aspergillusgenome.org/cgi-bin/compute/blast_clade.pl#Select_BLAST_Program). More specifically, the database “PROTEINS—translations of coding sequence (Protein)” (containing predicted protein sequence of all of the ORFs) was queried with default settings. The search for AN10216 homologs in Basidiomycota was carried out using the BLASTP algorithm at the NCBI website and by querying the non-redundant protein sequences in Basidiomycota (tax id: 5204) with default settings. E-values for the Aspergilli homologs ranged around 0 to 10^−170^, for the Basiomycota homologs around 10^−18^, and for plants around 10^−48^. Bits scores were larger than 200 in Aspergilli homologs and 80–200 in the other taxa. For the phylogenetic analysis, AN10216-type proteins were identified by blasting the AN10216 protein against proteins of restricted taxa. Matches were back-blasted against *Aspergillus* proteins and only those that matched AN10216 were used for phylogeny analysis. The phylogenetic tree was built with Phylogeny.fr [26] (available at http://www.phylogeny.fr/). The amino acids putatively involved in establishing protein–protein interactions were identified using the WD40-repeat Protein Structure Predictor (WDSP web server [27,28]; available at http://wu.scbb.pkusz.edu.cn/wdsp/). The 3D structure was obtained by homology modeling using the iTASSER and Phyre2 servers ([29,30]; http://www.sbg.bio.ic.ac.uk/phyre2 and http://zhanglab.ccmb.med.umich.edu/I-TASSER/).

### 4.2. Fungal Cultivation, Preparation of Gene Replacement Cassette and Transformation

The *A. nidulans* recipient strain is SAA.111. The strain was grown according to [31]. Gene replacement was carried out with the DJ-PCR method, as described in [32]: the cassette containing the selectable marker *riboB* (AN00670) [33] was inserted to replace the locus AN1556.2. Details of the primers used for the DJ-PCR are given in Table S4. Transformation was carried out as described in [30]. Diploids were obtained according to the method described in [34] using the strain yA2 [17].

### 4.3. Southern Blotting and PCR of Genomic DNA

Genomic DNA was extracted using the Qiagen DNeasy Mini Kit (Qiagen, Leusden, The Netherlands), analyzed for integrity by agarose gel electrophoresis and quantified using a NanoDrop ND-1000 spectrophotometer (Thermo scientific, Villebon-sur-Yvette, France). Ten µg of genomic DNA from the control (SAA.111) and transformed strains were digested with 20 units of BamHI and PstI at 37 °C, then resolved by electrophoresis and transferred to Hybond N+ membranes (GE Healthcare, Piscataway, NJ, USA), as described in [35]. Blotting and membrane cross-linking was performed as described in [36]. The hybridization probe (859 bp) was amplified using the primers WD Southern Fwd and WD Southern Rev (Table S4), using as template genomic DNA from the transformed strain and labeled with a digoxigenin (DIG) PCR labeling kit (DIG High Prime DNA Labeling and Detection Starter Kit II, Roche Diagnostics, Mannheim, Germany), according to the manufacturer’s instructions. The pre-hybridization and hybridization steps were carried out as described in [36]. Signals were detected with a chemiluminescent image analyser (GelDoc EZ imaging system, Biorad, Vienna, Austria).

PCRs of genomic DNA were carried out using ten ng of extracted DNA and the primers WD nested Fwd and WD nested Rev (Table S4). The Q5 Hot Start High-Fidelity 2X Master Mix (New England Biolabs, Leiden, The Netherlands) was used following the manufacturer’s instructions. The products were excised from the gel, purified using a gel extraction kit (Qiagen, Leusden, The Netherlands) and sequenced to verify the specificity of the amplicons.

### 4.4. Growth Tests

The sensitivity of the control and transformed strains to the cell wall-perturbing agents Congo Red (CR) and dichlobenil (DCB) was assessed as described in [9]. Additionally, sensitivity to osmotic stress (KCl 0.5 M) was analyzed on mycelia grown on solid minimal medium (MM) with the required supplements.

### 4.5. Optical, Confocal, Scanning Electron and Atomic Force Microscopy of A. nidulans Mycelia

Mycelia for optical and confocal microscopy were prepared as described in [9]. SEM analysis was performed on a Hitachi TM3030 tabletop microscope (Hitachi, Mannheim, Germany), after having collected the mycelium with a cut tip and having let it dry for a few minutes at room temperature. Atomic force microscopy (AFM) imaging was carried out as described in [9].

### 4.6. RNA Extraction, cDNA Synthesis and qPCR

RNA was extracted as described in [9]. Integrity was analyzed using the Agilent bioanalyzer, and RNA integrity numbers (RINs) were >8 for all the samples. The purity/concentration was assessed with a NanoDrop ND-1000 spectrophotometer (Thermo scientific, Villebon-sur-Yvette, France). RNA extraction, retrotranscription and qPCR were performed as described in [37]. The reactions were performed in technical triplicates. The PCR conditions were as described in [37]. The specificity of the amplified products was checked with a melting curve analysis. All of the qPCR amplicons were verified with sequencing, as previously described.

Five candidate reference genes were analyzed, namely rpl37 (AN04787), rpl3 (AN06202), actin (AN06542), CRP2 (AN05960) and TEF1 (AN02063). Ranking with geNormPLUS [38] indicated rpl37 and rpl3 as the most stable genes. Ten genes linked to cell wall glycan biosynthesis (described in [9]), together with AN10216 and the *bf* gene, a putative capsule polysaccharide synthase (*CPS1*, AN09069) and three cell wall sensors (*wscA* AN05660, *wscB* AN06927 and *pkcA* AN00106) were analyzed. The primers used have either been previously described [9] or listed in Table S4. The data were processed using the software qBasePLUS version 2.5 (Biogazelle, Ghent, Belgium) [39]. The calculated gene expression values are here indicated as “Normalized relative expression”. After log2 transformation of the normalized relative quantities exported from qBasePLUS, a one-way ANOVA was carried out with IBM SPSS Statistics (version 19, IBM SPSS, Chicago, IL, USA). A Tukey’s HSD was performed as post-hoc test. The normal distribution of the data was verified with a Kolmogorov–Smirnov test.

## 5. Conclusions

In conclusion, we provide evidence for the conserved genomic configuration chs-WDR gene in several fungi and WDR gene-bf gene in *Aspergillus* and present data suggesting their involvement in cell wall-related processes. Further functional analyses are necessary to shed light on the roles that FPWD and the bf protein play in cell wall biosynthesis in *A. nidulans*.

## Supplementary Materials

Supplementary materials can be found at http://www.mdpi.com/1422-0067/17/7/1031/s1.
