# Supplementary material for: A WDR Gene Is a Conserved Member of a Chitin Synthase Gene Cluster and Influences the Cell Wall in Aspergillus nidulans
Source: Int J Mol Sci. 2016 Jun 29;17(7):1031. doi: 10.3390/ijms17071031 (PMC4964407; doi:10.3390/ijms17071031)
Supplement: Supplementary file 1 [file ijms-17-01031-s001.pdf]

Supplementary Materials: A WDR Gene Is a Conserved Member of a Chitin Synthase Gene Cluster and Influences the Cell Wall in *Aspergillus nidulans*

Gea Guerriero, Lucia Silvestrini, Michael Obersriebnig, Jean-Francois Hausman, Joseph Strauss and Inés Ezcurra

Table S1. Locus numbers for *chs* and WDR genes in fungal species showing collinear *chs*-WDR genes. Asterisk indicates probable wrong annotation predicting the *chs* fused to the WDR gene.

Species	*chs* (Locus id)	WDR (Locus id)
Fusarium graminearum	FG10619.1	FG10616.1
Neurospora crassa	NCU09324	NCU09325
Penicillium chrysogenum	Pc12g02600	Pc12g02590
Byssochlamys spectabilis	GAD94253*	GAD94253*
Coccidioides immitis	CIMG_08766	CIMG_08764
Uncinocarpus reesii	UREG_02708	UREG_02705
Paracoccidioides lutzii	PAAG_04862	PAAG_04865
Arthroderma otae	MCYG_02610	MCYG_02611
Trichophyton verrucosum	TRV_03668	TRV_03670
Sclerotinia sclerotiorum	SS1G_04263	SS1G_04255
Marssonina brunnea	MBM_06183	MBM_06185
Pyrenophora tritici-repentis	PTRG_07569	PTRG_07571
Setosphaeria turcica	estExt_Genewise1.C_200184	estExt_Genewise1.C_200186
Bipolaris zeicola	e_gw1.170.42.1	e_gw1.170.30.1
Phaeosphaeria nodorum	SNOG_04257	SNOG_04254
Leptosphaeria maculans	LEMA_P024060.1	LEMA_P024040.1
Colletotrichum gloeosporioides	CGGC5_7101	CGGC5_7102
Verticillium albo-atrum	VDBG_05981	VDBG_05983
Chaetomium thermophilum	CTHT_0059710	CTHT_0059720
Podospora anserina	PODANSg5938	PODANSg5939
Myceliophthora thermophila	MYCTH_2296388	MYCTH_2296384
Aspergillus nidulans	ANID_01555	ANID_10216
Aspergillus versicolor	Aspve1_0079582	Aspve1_0123502
Aspergillus sydowii	Aspsy1_0143806	Aspsy1_0055743
Aspergillus flavus	AFL2G_00575	AFL2G_00574
Aspergillus oryzae	AO090005000579	AO090005000578
Aspergillus terreus	ATEG_07757	ATEG_07758
Aspergillus carbonarius	Acar5010_211936	Acar5010_510790
Aspergillus clavatus	ACLA_059050	ACLA_059060
Neosartorya fischeri	NFIA_098390	NFIA_098400
Aspergillus fumigatus	Afu8g05630	Afu8g05640
Aspergillus glaucus	Aspgl1_0125048	Aspgl1_0035139
Aspergillus wentii	Aspwe1_0119338	Aspwe1_0118846
Aspergillus niger	An09g02290	An09g02280
Aspergillus acidus	Aspfo1_0144304	Aspfo1_0051389
Aspergillus brasiliensis	Aspbr1_0032516	Aspbr1_0198343
Aspergillus tubingensis	Asptu1_0136299	Asptu1_0392309
Aspergillus zonatus	Aspzo1_0016743	Aspzo1_0133145
Aspergilluskawachii	Aspka1_0179037	Aspka1_0179039
Aspergillusaculeatus	Aacu16872_031285	Aacu16872_053227
Aspergillus ruber	gm1.7455_g	gm1.7454_g
Puccinia graminis	PGTG_10666	PGTG_10670
Cryptococcus gattii	CGB_F4460C	CGB_F4440C
Cryptococcus neoformans	CNF01610	CNF01630
Ustilago maydis	UM04290.1	UM04291.1

Table S2. *Arabidopsis* and poplar orthologs of FPWD with details on their tissue expression.

Gene	Expression
AT1G78070	not in database
AT1G36070	mature embryo, stem
Potri.002G094100, POPTR_0002s09470	root
Potri.005G168600, POPTR_0005s16680	xylem & root
AT1G55680	seed & pollen
AT3G13340	seed & other
Potri.011G168600, POPTR_0011s17160	root (catkin)
Potri.006G000500, POPTR_0006s00270	root & seedling
Potri.001G471800, POPTR_0001s47580	root (catkin)
AT5G56190	pollen
Potri.002G233700, POPTR_0002s23480	not in database
Potri.014G147400, POPTR_0014s14460	male catkin & root

Table S3. Fungal species showing conserved genomic association WDR-*bf* gene. Asterisk indicates probable wrong annotation predicting the WDR gene fused to the *bf* gene.

Organism	WDR (Locus id)	*bf* Gene (Locus id)
Aspergillus nidulans	AN10216	AN10219
Aspergillus versicolor	Aspve1_0123502	Aspve1_0079584
Aspergillus niger	An15g04470	An15g04480
Aspergillus acidus	Aspfo1_0061227	Aspfo1_0219095
Aspergillus carbonarius	Acar5010_510790	Acar5010_010551
Aspergillus brasiliensis	Aspbr1_0032637	Aspbr1_0198466
Aspergillus clavatus	ACLA_059060	ACLA_059070
Neosartorya fischeri	NFIA_098400	NFIA_098410
Aspergillus fumigatus	Afu8g05640	Afu8g05650
Aspergillus tubingensis	Asptu1_0048296	Asptu1_0156027
Aspergillus wentii	Aspwe1_0118846	Aspwe1_0045178
Aspergillus kawachii	Aspka1_0180599	Aspka1_0180600
Aspergillus sydowii	Aspsy1_0055743*	Aspsy1_0055743*
Aspergillus flavus	AFL2G_00574	AFL2G_00573
Aspergillus oryzae	AO090005000578	AO090005000577
Aspergillus aculeatus	Aacu16872_053227	Aacu16872_044768
Aspergillus glaucus	Aspgl1_0035139	Aspgl1_1516651
Aspergillus zonatus	Aspzo1_0133145	Aspzo1_0097766
Penicillium chrysogenum	Pc12g02590	Pc12g02580
Coccidioides immitis	CIMG_08764	CIMG_08762
Uncinocarpus reesii	UREG_02705	UREG_02704
Arthroderma otae	MCYG_02611	MCYG_02612
Aspergillus ruber	gm1.7454_g	estExt_Genemark1.C_310069
Puccinia graminis	PGTG_10670	PGTG_10672

Table S4. Sequence of primers used in the study.

Name	Sequence (5′→3′)
**WD Southern Fwd**	TTGCCGCGTGAGCCTGAAGTCTTGCCATTT
**WD Southern Rev**	TGTGGCGAGATTTGTTGAAGTAACCGTTCT
**WD Upstream Fwd**	GAGGGTGGGATAAGGCGAAAGAGGAGTT
**WD Upstream Rev**	GTCTTGTGAACGAAACCGCGAAGATTCC
**WD Downstream Fwd**	GCTGGCTAGAGTTGCAGAACGATCTGCT
**WD Downstream Rev**	AAACCCGCACCTCCACCTCCACCTCCAG
**Ribochimera Fwd**	GGGGAATCTTCGCGGTTTCGTTCACAAGACCGTACGTAGTGTAGATTCA GGCACATTGAAGCG
**Ribochimera Rev**	CCAGCAGATCGTTCTGCAACTCTAGCCAGCCTGCCATGACTACTAGGT GGTGCTATCATT
**WD nested Fwd**	CTACGTCGTTGTTTCTTCAATCATCAAA
**WD nested Rev**	TTTGAGGTGCGTCTGGATCGGGAGCTAG
**CPS1 Fwd**	ACGATCGAAACCATCCTGAC
**CPS1 Rev**	CGGCTTAGCATTTCTTCAGC
**rpl3 Fwd**	TTCCTCGCAAGACTCACAAG
**rpl3 Rev**	TTGTGGTTGCAAGAGGTACG
**act Fwd**	ATTGAGCACGGTGTTGTCAC
**act Rev**	GTTGGACTTGGGATTGATGG
**rpl37 Fwd**	CGCCACAACAAAACTCACAC
**rpl37 Rev**	TCTCGCTCCAGTTGTACTTGC
**pkcA Fwd**	AACCGGTCTTCTGACAATGC
**pkcA Rev**	ACCTGGTTGCCTTTGTTCTG
**wcsB Fwd**	CATCTTCTTCCTCGCAAAGC
**wcsB Rev**	AAGATCCGTTGGACAAGGTG
**wcsA Fwd**	TCATTGGTGTTGTGGGTCTG
**wcsA Rev**	AGTCTTGCGACATGGGTTTG
**FPWD Fwd**	ACCATGAAAACGGCTGTAGG
**FPWD Rev**	TTCCACTCCCTTGGTTTCAG
**bf Fwd**	TTCCTCCGAAGACTCCAATC
**bf Rev**	TCCTCGTCGATGTCATCTTG

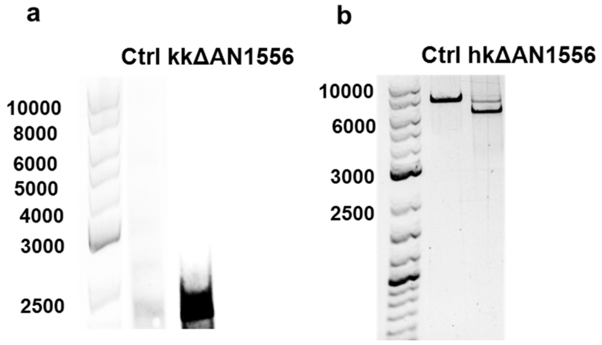

Figure S1. Molecular characterization of hkΔAN1556: (**a**) Southern blotting analysis; and (**b**) PCR on SAA.111 (Ctrl) and hkΔAN1556. The size of the band hybridizing with the probe in the transformed line is 2572 bp. The sizes of the PCR bands are 7408 and 6204 bp.

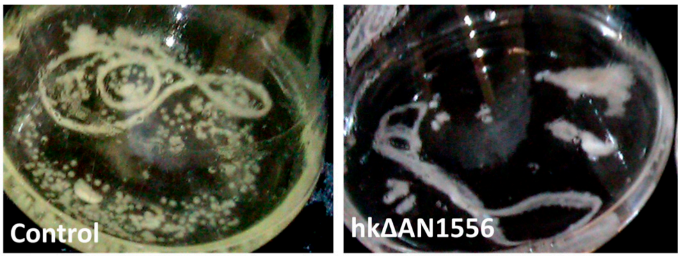

Figure S2. Phenotypes in liquid culture of control and hkΔAN1556 mycelia. The formation of big irregular clumps is evident in hkΔAN1556.

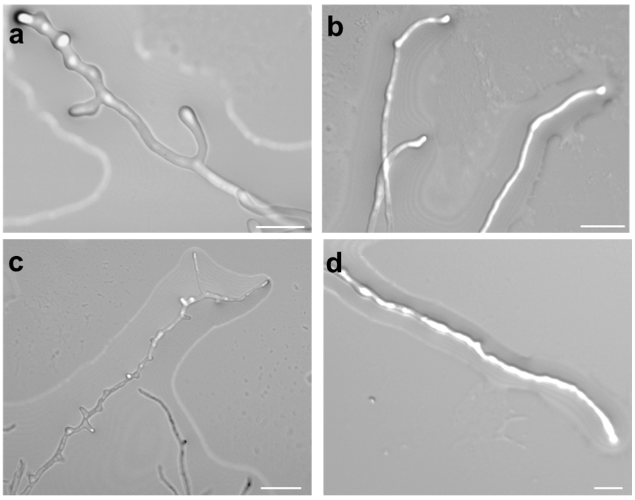

Figure S3. Differential interference contrast pictures of the four diploids showing the presence of non-straight, vacuolated hyphae: (**a**) diploid D1; (**b**) diploid D2; (**c**) diploid D3; and (**d**) diploid D4. Scale bars refer to 10 μm.

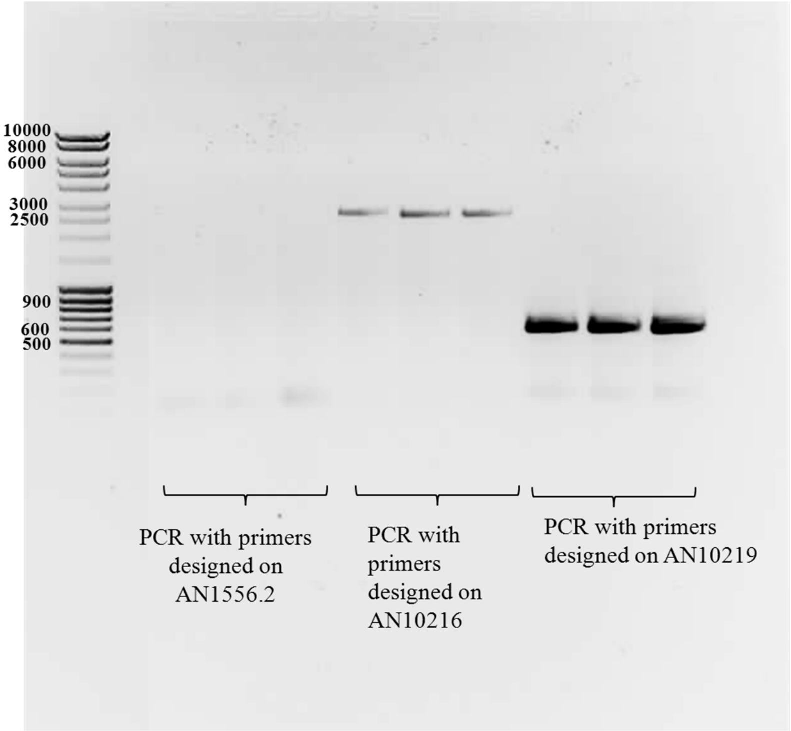

Figure S4. PCR on cDNA confirming the reannotattion of the *FPWD* locus, AN10216. The primers are designed to amplify the full-length genes. The PCR product is 2484 bp for AN10216 and 543 bp for AN10219.

```
ANID_10219            1 ------------------------------------------------------------
CIMG_08762            1 ------------------------------------------------------------
estExt_Genemark1      1 DGPPQSSGYGGGGSYGGPSYGGSSGGHGGHGGPPHGSSGGYGGPPQSGGYGG---GDSYG
MCYG_02612            1 ------------------------------------------------------------
UREG_02704            1 ------------------------------------------------------------
Pc12g02580            1 -------GYGSN--------------------------EGYGNQNQ--GYGN-NNNNSYG
Aspzo1_0097766        1 ------------------------------------------------------------
AFL2G_00573           1 NDRPS-QGYGGD-SYN--------------DRPSHGNQGGYNDRPS-SGYGG----DSYG
Aspve1_0079584        1 ------------------------------------------------------------
Aspgl1_1516651        1 -----------------------------------------------GGYGG---GDSYG
An15g04480            1 ------------------------------------------------------------
Aspfo1_0219095        1 ------------------------------------------------------------
Acar5010_010551       1 ------------------------------------------------------------
Aspbr1_0198466        1 ------------------------------------------------------------
ACLA_059070           1 ------------------------------------------------------------
NFIA_098410           1 ------------------------------------------------------------
Afu8g05650            1 ------------------------------------------------------------
Asptu1_0156027        1 ------------------------------------------------------------
Aspwe1_0045178        1 ------------------------------------------------------------
Aspka1_0180600        1 ------------------------------------------------------------
AO090005000577        1 NDRPS-QGYGGD-SYN--------------DRPSHGNQGGYNDRPS-SGYGG----DSYG
Aacu16872_044768      1 ------GGYGGEQSYG--SQGGSYGG-GPQGGSYGGSQGGYGGEQG-GSYGGGPQGGSYG
consensus             1
ANID_10219            1 ------------------------------------------MS-LQNILVDAV---K--
CIMG_08762            1 ------------------------------------------MSNYSRPPNPAEYG-GGG
estExt_Genemark1     58 G----GSSGGYGGPP-----------------------QSSGYG-GGDSYGGPSYG-G--
MCYG_02612            1 ------------------------------------------MS-YNDTQHRGEAA-G--
UREG_02704            1 ------------------------------------------------------------
Pc12g02580           25 G-----SGGGYGNSN--------------------------------DAYGNQNQG-N--
Aspzo1_0097766        1 ------------------------------------------------------------
AFL2G_00573          40 GRPSHGNQGGYNDRP------------------------SSGYG-GDSYNDRPSHG-N--
Aspve1_0079584        1 ------------------------------------------MS-FQNIIAGAV---K--
Aspgl1_1516651       11 G----PSSGGHGGPP-----------------------QSSGYG-GGDSYGGPSYG-G--
An15g04480            1 ------------------------------------------MS-FSNLISSALEQ-A--
Aspfo1_0219095        1 ------------------------------------------MS-FSNLISSALEQ-A--
Acar5010_010551       1 ------------------------------------------MS-FSNLISSAIEQ----
Aspbr1_0198466        1 ------------------------------------------MS-FSNLISSAIEQ-A--
ACLA_059070           1 ------------------------------------------MS-LSNFIQDAISGAT--
NFIA_098410           1 ------------------------------------------MS-FSNMIQDALSG-H--
Afu8g05650            1 ------------------------------------------MS-FSNIIQDALSG-H--
Asptu1_0156027        1 ------------------------------------------MS-FSNLISSALEQ-A--
Aspwe1_0045178        1 ------------------------------------------------------------
Aspka1_0180600        1 ------------------------------------------MS-FSNLISSALEQ-A--
AO090005000577       40 GRPSHGNQGGYNDRP------------------------SSGYG-GDSYNDRPSHG-N--
Aacu16872_044768     51 G----GPQGGYGGPQGGYGGEQGGYQQQHHHQQHHQQQQGGYS-EGGSYGGGPQG-G--
consensus            61                                           ms y n l  av g
ANID_10219           13 -SHFDKDDDKD-------------------------------------------------
CIMG_08762           18 SSGANYYDPRA-DFSSQS----------QPYQQQ-------SQYQG--------------
estExt_Genemark1     87 -SSGSHGGPSH-GSS--------------------------GGYGG--------------
MCYG_02612           15 -----YYNP---------------------------------------------------
UREG_02704            1 ------------------------------------------------------------
Pc12g02580           45 -YG---RQESH-G----------------------------GGYG---------------
Aspzo1_0097766        1 ----------------------------MSY----------NGYNG--------------
AFL2G_00573          72 -QGGYNDRPSS-GYGGDSYNDRPSHGNQGGYNDRPSHGGNQGGYGGGSYNDTSYNAPPP
Aspve1_0079584       13 -DHFNQDSGHS-GNQGG-------------------------------------------
Aspgl1_1516651       40 -SSGSHGGPSH-GSSGGY-------------------GNSSGGYGG--------------
An15g04480           15 -TSHS---------SGG------------------------SG-----------------
Aspfo1_0219095       15 -TSGSHSSSSG-GYSGG--------G---------------GGYGG--------------
Acar5010_010551      14 -------------GLSGG---------------------------G--------------
Aspbr1_0198466       15 -TSGNHNSSSGGGYGGS------------------------GGYGG--------------
ACLA_059070          16 -GHNNENNSSY-GNNPSYGN----------------------------------------
NFIA_098410          15 -GHSNNNNNRH-DND---------------------------------------------
Afu8g05650           15 -SHSN-NNSRH-DNN---------------------------------------------
Asptu1_0156027       15 -TSGSHSSSSG-GYSGG------------------------GGY-G--------------
Aspwe1_0045178        1 ------MASND-YYNQG------------------------NSYGG--------------
Aspka1_0180600       15 -TSGSHSSSSG-GYSGG------------------------GGYGG--------------
AO090005000577       72 -QGGYNDRPSS-GYGGDS-------------------------------YNDTSYNAPPP
Aacu16872_044768    103 -SYG--GEPSY-GSQGSY-------------------G---GGSNN--------------
consensus           121  s       s  g  g                          gy g
```

Figure S5. *Cont.*

```
ANID_10219           23 -----------------------------------------DLKPALSHASA-NASS---
CIMG_08762           46 G-----SGGY-NYDPAFS---------------------N-AVKQAKYHNSG-REDD---
estExt_Genemark1    105 S-----SQGH-GRHDSDS----------D----------D-DFNPALSHAQS-SGGS---
MCYG_02612           19 -------GDL-SLNQ--D---------------------Q-AVRQAQYHKNG-REDH---
UREG_02704            1 ------------------------------------------------------------
Pc12g02580           57 N-----NSSS-HHSNYND----------D----------N-DYSGAAMHAQE-HHSN---
Aspzo1_0097766        9 G--GA-SASY---YDNNN----------D--FS------D-ELRGAAQHAER-GHSA---
AFL2G_00573         130 S-----HGGY-HNSNPSS----------H-GFSD-----S-DVSPALAHAQQHSDSS---
Aspve1_0079584       28 ----------------------------N----------QFDLNGAASHAST-H-SS---
Aspgl1_1516651       65 S-----SQGH-GHHDSDS----------D----------D-DLNPALSHAQS-SGGS---
An15g04480           24 ------SGSY-D---------------------------P-EFSSAQHHAQA-HESS---
Aspfo1_0219095       36 G-----GGSY-D---------------------------P-EFSSAQHHAQS-HESS---
Acar5010_010551      20 N-----NSNY-D---------------------------P-EFSSAHSHAQS-HHASYNQ
Aspbr1_0198466       36 NYGGNESGSY-D---------------------------P-EFSSAQHHAQS-HESS---
ACLA_059070          34 ------NPSY-STNPPPSHNPNNSAPFAN----------D-DFNPALRYAES-HSNT---
NFIA_098410          28 ----------------------------N----------D-DFNPAVRYAES-HTNT---
Afu8g05650           27 ----------------------------Y----------D-EFNPAVRYAES-HANT---
Asptu1_0156027       34 N-----SGSY-D---------------------------P-EFSSAQHHAQS-HESS---
Aspwe1_0045178       16 G---H-GHSYGD---------------------------D-DFSSAAHHAQA-HHES---
Aspka1_0180600       35 G-----GGSY-D---------------------------P-EFSSAQHHAQS-HESS---
AO090005000577       99 S-----HGGY-HNSNPSS----------H-GFSD-----S-DVSPALAHAQQHSDSS---
Aacu16872_044768    123 N-----EGNY-GHNPPHQ----------NPSYGGGRPSYD-DVASATSHAESHSAGT---
consensus           181        g y                               df  a  haqs h ss

ANID_10219           38 -------------------------------E--D--SNLFSQALSFINQRKSEG-----
CIMG_08762           74 ----------DE-------------------D--DEESGFFKKALSFISEHKDRF-----
estExt_Genemark1    134 -------------------------------G--D--SSLFSSALSFIKDQKKKK-----
MCYG_02612           43 ----------ED-------------------D--D--SSLFSKAMSFLGDNKEKL-----
UREG_02704            1 ------------------------------------------------------------
Pc12g02580           86 -------------------------------E--D--TSLFSSALNFLKDRKNND-----
Aspzo1_0097766       40 ------------------P------------D--S--SSLFGEAVSFIQGRASEYMSGGG
AFL2G_00573         164 -------------------------------S--D--SSLFSTALNFIKDKQGRS-----
Aspve1_0079584       45 -------------------------------E--D--SSLFSQALKFVQERNS-G-----
Aspgl1_1516651       94 -------------------------------G--D--SSLFSSALSFIKDQKKKK-----
An15g04480           45 -------------------------------G--D--SSLFSSALGFLSDRKSQY-----
Aspfo1_0219095       58 -------------------------------S--D--SSLFSSALGFLSDRKSQY-----
Acar5010_010551      45 GGYGNQGGYSDDQGRDSYPSQGGGGGGSGSGS--S--SDLFSTALSFLSSRKSQY-----
Aspbr1_0198466       63 -------------------------------S--D--SSLFSSALSFLSDRKNQY-----
ACLA_059070          72 -------------------------------SSND--SSLFTSALSFLNENKHRF-----
NFIA_098410          45 -------------------------------S--D--PSLFTSALSFLNENKHRL-----
Afu8g05650           44 -------------------------------S--D--SSLFTSALSFLNENKHRL-----
Asptu1_0156027       56 -------------------------------S--D--SSLFSSALGFLSDRKSQY-----
Aspwe1_0045178       40 ---------------------------S---E--D--SSLFSSAISFIKDRQSQG-----
Aspka1_0180600       57 -------------------------------S--D--SSLFSSALGFLSDRKSQY-----
AO090005000577      133 -------------------------------S--D--SSLFSTALNFIKDKRGRS-----
Aacu16872_044768    163 -------------------------------G--S--SDLFQKALGYITNRHSSS-----
consensus           241                                   d  sslfssalsfl drk

ANID_10219           58 ---V-QD--DIDEEQAVNAHRRYEQG-------------GNMDSKDFGAGAALQALKMF-
CIMG_08762           98 -----GQE-DIDEQQVVGAHQALYGG-GQQQQQQ-QQAERKHDADFLGNGAALQALKMF-
estExt_Genemark1    154 ---DYKND-EINDNEMVSAHQSLYGS--G------GSDDRKHDSNSVGMGAAMQALKMF-
MCYG_02612           65 -----GRE-DIDEQKVVNSHQKLYGD-------V-DDRNEKHGAESLGSGAAMQALKMFL
UREG_02704            1 ---------------MVGAHQALYGG-----QHE-GGDRGKVDADFLGSGAALQALKMF-
Pc12g02580          106 ---D--DN-DVDEEKAANAHQAMYGS---------GSSNEKHDSNTVGAGAAMQALKMF-
Aspzo1_0097766       66 GGGS-SGP-AVDENQMVQAHQAVYGQ--G-S----QGGGQQHSSETLGAGAAMQALKMF-
AFL2G_00573         184 ---S--SP-DIDEDQMVQSHQQLYND---------NDSSKAHDSNSLGAGAAMQALKMF-
Aspve1_0079584       64 ---S-DH--DVDEEHAVNSHRRYEQG-------------GNMDSRDMGAGAAMQALKMF-
Aspgl1_1516651      114 ---DYKND-EVNDDEMVSAHQSLY-N--G------GSDDRKHDSNSVGMGAAMQALKMF-
An15g04480           65 ---S-EQP-DIDEEHLVQSHQQLYNG--GGG-GY-GQEQQQHDSKSLGAGAAMQALKMF-
Aspfo1_0219095       78 ---S-EQP-DIDEEHLVQSHQRLYNG--G------GE-QQQHDSSSLGAGAAMQALKMF-
Acar5010_010551      96 ---E-EQP-DVDEEHMVQSHQALYNN---------QDEGQQHDSKSLGAGAAMQALKMF-
Aspbr1_0198466       83 ---S-EQP-DIDEEHLVQSHQALYNG--GGGGGY-GGGEQQHDSKSLGAGAAMQALKMF-
ACLA_059070          94 ---S-DDRADLNEADVVRAHQSLYNN-NGGNE---GQGQGRHDAGSLGAGAALQALKLF-
NFIA_098410          65 ---S-NDN-DINEQEMIHAHQSLYGG---------GESERRHDSSSVGAGAAMQALKMF-
Afu8g05650           64 ---S-NDN-DINEQEMIHAHQSLYDG---------RDSERRHDSSSVGAGAAMQALKMF-
Asptu1_0156027       76 ---S-EQP-DIDEEHLVQSHQRLYNN--GGG--YGGEQQQQHDSSSLGAGAAMQALKMF-
Aspwe1_0045178       61 ---S-HHE-EIDENEVLSSHQSLYNN---------EDDGRSHDSKSLGSGAALQALKMF-
Aspka1_0180600       77 ---S-EQP-DIDEEHLVQSHQRLYNG--G------GEQQQQHDSSSLGAGAAMQALKMF-
AO090005000577      153 ---S--SP-DIDEDQMVQSHQQLYND---------NDSSKAHDSNSLGAGAAMQALKMF-
Aacu16872_044768    183 ---EDDDD-DIDESQMVQSHQAVYNSHEG------AGQQQQHDSKTLGAGAAMQALKMF-
consensus           301    s     didee vvnsHq lyn          g     hds slGaGAAmQALKmF
```

Figure S5. *Cont.*

```
ANID_10219          98 ---NSSSG--QETGG------GK-DKNAFIGMAMAQAAKMWEEKA-GKG-EASGDKQSAI
CIMG_08762         149 ---TSGEG--QQS-Q--TG--GH-DQNKLIGLAMAQAGKLWDQQN-QQG-NVATDKQSVI
estExt_Genemark1   201 ---TSSDS--GSG-SGSSS--GGMDKNKLIGIAMSQAGDLWEKKN-SGG-QASGDKQSAV
MCYG_02612         111 NDGNKNQG--QNQ-S--QG--GG-DQNKLIGMAMAQAGKMWEQKN-QEG-RVDTDKQTAV
UREG_02704          39 ---SSGEG--RQS-Q--SG--GH-DQNKLIGLAMAQAGKLWDQQN-QQG-QVATDKQSAI
Pc12g02580         150 ---AGGNE--SSG-SSG----GGMDKNKLIGLAMAQAGKLWDEKNGSGG-DVSGDKQSAV
Aspzo1_0097766     116 ---GSG----SSG-S------SG-SMNEFIGMAMSQAGNLWDQHS-ASG-SVAGSKQSAI
AFL2G_00573        228 ---SSGQS--GGS-S------GG-DQNAFIGMAMSQAAKLWEQKN-SSG-NVTDDKQSAV
Aspve1_0079584     104 ---NSGSG--GASGG------GQ-DKNAFIGLAMAQASKMWEEKN-GKG-EASGDKQSAV
Aspgl1_1516651     160 ---SSSDS--GSG-SGSSSSSGGMDKNKLIGIAMSQAGDLWEKKN-SGG-QASGDKQSAV
An15g04480         115 ---TSG----GGG-S------SG-DKNEFIGMAMAQASKLWEEKS-GSG-NVSGDKQSAV
Aspfo1_0219095     123 ---TSGG---GGS-S------GG-DKNEFIGMAMAQASKLWEEKA-GSG-NVSGDKQSAV
Acar5010_010551    141 ---TSG----SGG-S------SG-DKNEFIGMAMAQASKLWDQKS-GSG-SVAGDKQSAI
Aspbr1_0198466     134 ---TSGGGSSGSS-S------GG-DKNEFIGMAMAQASKLWDQKS-GSG-NVSGDKQSAI
ACLA_059070        145 ---TTSSE--GEK-S------GM-SKDAFIGLAMAQAKRLFEEKE-GKG-EVSGDKQSAI
NFIA_098410        110 ---TSSSE--GEK-S------GM-DKNAFIGLAMAQAKKMFEEKE-AKG-EVNGDKQSAI
Afu8g05650         109 ---TSSSE--GEK-S------GM-DKNAFIGLAMAQAKKMFEEKE-AKG-EVNGDKQSAI
Asptu1_0156027     126 ---TSGGG--GGS-S------GG-DKNEFIGMAMAQASKLWEEKA-GSG-NVSGDKQSAV
Aspwe1_0045178     106 ---TGGGGSSGGS-G------GM-DKNELIGLAMSQAGKLWEQKQ-SSGGNVSGDKQSAI
Aspka1_0180600     123 ---TSGGG--GGS-S------GG-DKNEFIGMAMAQASKLWEEKA-GSG-NVSGDKQSAV
AO090005000577     197 ---SSGQS--GGS-S------GG-DQNAFIGMAMSQAAKLWEQKN-SSG-NVTDDKQSAV
Aacu16872_044768   232 ---TSGSG--GSG-SSSGS--GG--KNEFIGLAMAQASKLWEEKS-SSG-LASGDKQSAI
consensus          361    tsg    g t s      gg dkn fIGmAMaQAaklweek  gsG nvsgdKQsai

ANID_10219         144 NQAAEMAFKMYLKSQMSG----SEGTGG--------------------------------
CIMG_08762         196 NSAAKMALKMYLKNQAGGGGGALGGLGG--------LGG---------------LASGVS
estExt_Genemark1   251 NSAAEMALKMYMKSNGSG----SQGTGG--------------------------------
MCYG_02612         161 NMAAQYALKMYLKGQMGGGSTGMGGLGGGLALISSALGGGGGHQQQQQSGLAALAGAL-
UREG_02704          86 NSAAKMALKMYLKNQSGG---KLGGVGG--------LGG---------------LAGSL-
Pc12g02580         199 NSAAEMALKMYMKNQMSG----GGSSGG--------------------------------
Aspzo1_0097766     159 NSAAEMAMKMYMKSGGSG----LGGTGG--------------------------------
AFL2G_00573        273 NKAAEMAMKMYMKSQGSG----SSGSGG--------------------------------
Aspve1_0079584     150 NQAAEMAFKMYMKSEGSG----SSGTGG--------------------------------
Aspgl1_1516651     212 NSAAEMALKMYMKGNGSG----SQGTGG--------------------------------
An15g04480         158 NSAAEMAFKMYMKSQGSG----SSGTGG--------------------------------
Aspfo1_0219095     167 NSAAEMAFKMYMKSQGSG----SSGTGG--------------------------------
Acar5010_010551    184 NSAAEMAFKMYMKSQGSG----SSGTGG--------------------------------
Aspbr1_0198466     181 NSAAEMAFKMYMKSQGSG----SSGTGG--------------------------------
ACLA_059070        190 NSAAEMALKMYMKSQGGG----MSGTGG--------------------------------
NFIA_098410        155 NAAAEMALKMYLKSGGGG----MAGTGG--------------------------------
Afu8g05650         154 NAAAEMALKMYLKSGGGG----MTGTGG--------------------------------
Asptu1_0156027     171 NSAAEMAFKMYMKSQGSG----SSGTGG--------------------------------
Aspwe1_0045178     154 NNAAEMAMKMYMKSQGGG----IGGTGG--------------------------------
Aspka1_0180600     168 NSAAEMAFKMYMKSQGSG----SSGTGG--------------------------------
AO090005000577     242 NKAAEMAMKMYMKSQGSG----SSGSGG--------------------------------
Aacu16872_044768   280 NQAAEMALKMYMKSQGSG----SSGTGG--------------------------------
consensus          421 NsAAemAlKMYmKsqgsG    ssgtGG
```

Figure S5. *Cont.*

```
ANID_10219          168 ---------------P------G-G----LMSLASKFL-K--------
CIMG_08762          233 GGG------------Q--S---G-G-SG-LIGLAKKFL----------
estExt_Genemark1    275 ---------------PSSSGGAS-G----LLSLASKFM----------
MCYG_02612          220 -GGSGGQQQQQQQQQQ--S---G-A-SN-LLNMASKLL-CRGCQVVTY
UREG_02704          119 -GG------------Q--S---G-G-SG-LFDLAKKFF----------
Pc12g02580          223 ---------------G---------G----LMSLASKFL----------
Aspzo1_0097766      183 ---------------P------A-G----LMSLASKFL----------
AFL2G_00573         297 ---------------P--------A----LMNLAAKFLSK--------
Aspve1_0079584      174 ---------------P------S-G----LMGLASKFL-K--------
Aspgl1_1516651      236 ---------------PSSSGGAS-G----LLSLASKFM----------
An15g04480          182 ---------------P------A-A----LMSLASKFL----------
Aspfo1_0219095      191 ---------------P------G-A----LMSLASKFL----------
Acar5010_010551     208 ---------------P------A-G----LMSLASKFL----------
Aspbr1_0198466      205 ---------------P------A-A----LMSLASKFL----------
ACLA_059070         214 ---------------P------G-G----LLSLASKFM----------
NFIA_098410         179 ---------------P------GGG----LLQLASKFL----------
Afu8g05650          178 ---------------P------G-G----LLQLASKFL----------
Asptu1_0156027      195 ---------------P------G-A----LMSLASKFL----------
Aspwe1_0045178      178 ---------------P------G-GGASGLLSLASKFL----------
Aspka1_0180600      192 ---------------P------G-A----LMSLASKFL----------
AO090005000577      266 ---------------P--------A----LMNLAAKFLSK--------
Aacu16872_044768    304 ---------------P------A-G----LLSLASKFL----------
consensus           481                p      g g    LmslAsKfl
```

Figure S5. Alignment of Bf proteins from different ascomycetes. The alignment was done with Boxshade (www.ch.embnet.org/software/BOX_form.html). Black shading, identical amino acids; gray shading, similar amino acids.
